# Progressively confluent monkeypox lesions with necrosis

**DOI:** 10.1093/omcr/omac153

**Published:** 2023-01-18

**Authors:** Kolton Smith, Kelly Tyson, Raeesa Hossain, Alexandra Young, Jillian Melnick

**Affiliations:** Dept. of Internal Medicine, Lenox Hill Hospital, New York, NY, USA; Dept. of Internal Medicine, Lenox Hill Hospital, New York, NY, USA; Dept. of Internal Medicine, Lenox Hill Hospital, New York, NY, USA; Dept. of Internal Medicine, Lenox Hill Hospital, New York, NY, USA; Dept. of Internal Medicine, Lenox Hill Hospital, New York, NY, USA

## Abstract

Human monkeypox virus became a burgeoning global health issue when outbreaks were identified in over 100 countries beginning in early 2022. We describe the case of a 38-year-old male with acquired immunodeficiency syndrome who presented one month after the development of painful anal lesions, subsequently confirmed to be monkeypox. The patient was unsuccessfully treated outpatient with multiple courses of oral tecovirimat before presenting to the emergency department for continual lesion progression. Given his AIDS, the patient was at-risk for poor response to oral treatment due to the potential for malabsorption from disruptions in his gut microbiome as well as inability to consume the recommended 25 grams of fat per dose needed for absorption. The identification of patients at-risk for severe disease is imperative as this population may be better suited for intravenous tecovirimat treatment due to the difficult parameters required for optimal absorption of oral therapy.

## INTRODUCTION

Human monkeypox virus (MPXV), an orthopoxvirus endemic to Central and West Africa, has been identified in 82 nonendemic countries with over 72 000 confirmed cases since May 2022 [1]. Though most commonly contracted through person-to-person contact, MPXV can also be transmitted through fomites and/or large respiratory droplets from infected persons [2]. Data from the current outbreaks indicate cases are mostly among men who have sex with men (MSM). In published cohorts of affected individuals, the proportion of patients who have human immunodeficiency virus (HIV) ranges from 36 to 42% [3].

Initial symptoms of infection with MPXV include fever, chills and myalgias, followed by a characteristic pox-like painful rash that evolves through several stages: macule, then papule, then vesicle and finally pseudo-pustule before crusting over. Severe disease manifestations include sepsis, encephalitis, secondary bacterial infection, ocular involvement and oral/pharynx/anogenital lesions that can develop strictures [4].

Antiviral treatment with tecovirimat, a p37 inhibitor, is indicated for specific populations including immunocompromised individuals, patients with concomitant infections, and those with indications of severe disease. Oral as well as intravenous (IV) formulations of tecovirimat are available for use in patients with MPXV [5].

## CASE REPORT

A 38-year-old male with acquired immunodeficiency syndrome (AIDS), a CD4 count of 30 and viral load of 58, presented to the hospital with a one-month history of paroxysmal fevers and worsening necrosis at the site of known monkeypox lesions along the buttocks. Six weeks prior, the patient had unprotected sex with another male. Days after the encounter, the patient developed painful perianal lesions. Polymerase chain reaction (PCR) assay of swabs from the anal lesions were positive for MPXV (PCR-based monkeypox (orthopoxvirus) DNA assay developed by the CDC, performed at LabCorp). At the time, the patient had a CD4 count of 18 (Helper T-Lymphocyte Marker CD4 testing Kit, LabCorp), a viral load of 114 467 and was started on the antiretroviral therapy. The patient was unsuccessfully treated with two courses of 600 mg oral tecovirimat every 12 hours for 14 days, experiencing worsening of the lesions through attempted therapy. The patient continued to develop novel lesions while his old lesions began to necrose and coalesce.

**Figure 1:**
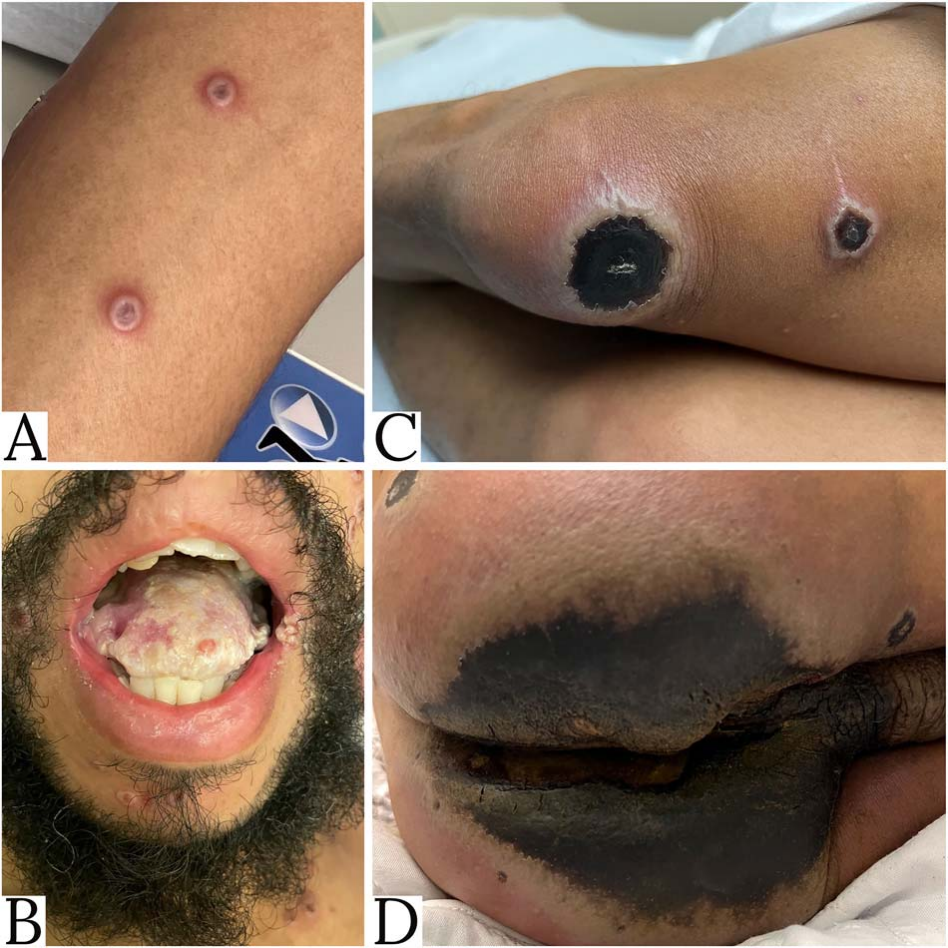
This figure demonstrates newly developing monkeypox lesions along the left forearm and in the mouth (Panels **A** and **B**) as well as confluence and necrosis of weeks-old lesions along the right knee and buttocks/anal region extending to the scrotum (Panels **C** and **D**).

In the emergency department, his vitals were T-98.9F, HR-94 bpm, BP-135/89 mmHg, RR-18, O2 sat-96% on room air. Upon review of systems, the patient endorsed watery diarrhea for one month. Physical examination exhibited newly developing monkeypox lesions along the left forearm and in the mouth superimposed on oral thrush (Fig. 1A and B) as well as confluence and necrosis of week-old lesions along the left knee and buttocks/anal region extending to the scrotum (Fig. 1C and D).

Labs were significant for leukocytosis with neutrophilic predominance, mild hyponatremia, prolonged coagulation studies and elevated inflammatory markers (Table 1). Lactate was normal, and gastrointestinal PCR panel was positive for Giardia, Shigella and Enterotoxigenic *E. Coli*. Abdominal and pelvic computed tomography showed distal rectal and perianal wall thickening, bilateral inner gluteal cellulitic changes with no drainable fluid collection, and reactive mesorectal and inguinal lymph nodes. The surgical service was consulted and determined there was no acute indication for debridement of the wounds.

**Table 1:** Lab-work upon initial presentation to the emergency department

Lab-work	Result
White blood cell count (K/μL)	15.5
Neutrophil # (K/μL)	11.8
Hemoglobin (g/dL)	12.0
Sodium	130
Partial thromboplastin time (s)	27.2
INR	1.31
Erythrocyte sedimentation rate (mm/h)	65
C-reactive protein (mg/L)	151.3
Lactate (mmol/L)	1.7

Following resuscitation with intravenous normal saline, the patient was started on intravenous tecovirimat 200 mg IV every 12 hours. In addition, he was started on Piperacillin/Tazobactam IV and Vancomycin IV for sepsis in the setting of suspected bacterial superinfection, Trimethoprim/Sulfamethoxazole orally for pneumocystis pneumonia prophylaxis, and Fluconazole IV oral and esophageal thrush. The patient showed clinical improvement after the first few days of intravenous tecovirimat and was started on antiretroviral therapy in the form of Biktarvy 1 tab daily. Unfortunately, after two weeks of improvement, the patient developed septic shock from multi-pathogen superinfection and died after a one-month hospitalization.

## DISCUSSION

The decision to treat with tecovirimat was clear as it is the only antiviral medication approved for use in MPXV in the USA. Early data suggest that early treatment is crucial to prevent viral spread and patient decompensation [6]. Tough intravenous tecovirimat is not realistic in every patient with AIDS given the multiple barriers to IV administration, in particularly high-risk patients it might be life-saving when compared to oral therapy.

The effectiveness of tecovirimat in the treatment of MPXV is largely influenced by the individual’s ability to consume and absorb the necessary amount of fat, as tecovirimat requires 25 grams of nutritional fat for optimal absorption into the bloodstream [5, 7]. In actively infected patients, it may be difficult to consume such high fat content twice daily due to decreased appetite or poor food availability. Beyond these factors, individuals with HIV/AIDS and diarrhea commonly have abnormal intestinal fat absorption at baseline [8]. This is usually due to infection with diarrhea-causing organisms, which themselves cause malabsorption via villous atrophy, increased intestinal permeability and rapid small bowel transit [8, 9]. Our patient, who presented with several diarrheal illnesses, likely had an inability to properly absorb dietary fat. It is also possible that our patient had some degree of HIV enteropathy, which is a direct HIV-mediated and indirect immune-mediated injury to the GI tract mucosa [10]. Beyond the physiological barriers to adequate absorption of oral tecovirimat, our patient also had painful MPXV lesions of the tongue along with severe oral candida that reduced his ability to tolerate any oral intake (Fig. 1B).

As we continue to battle the current MPXV outbreak, it is crucial to ensure that we use tecovirimat wisely. Clinicians must consider the best route of administration for each individualized patient by understanding risk-factors for malabsorption and practical barriers to ingesting the recommended amount of fat for optimal bioavailability of the oral formulation. Patients with HIV should have an up-to-date CD4 count and when indicated, we feel it is reasonable to screen patients for diarrheal pathogens and incorporate this information into treatment decisions. Patients undergoing oral tecovirimat therapy must also be educated thoroughly on the dietary fat recommended for proper absorption of the medication. Our case demonstrates that patients who are severely immunocompromised, such as those with AIDS and active diarrheal illness, would likely receive greater benefit from IV tecovirimat versus oral therapy.
